# Validation of the Apple Watch for Heart Rate Variability Measurements during Relax and Mental Stress in Healthy Subjects

**DOI:** 10.3390/s18082619

**Published:** 2018-08-10

**Authors:** David Hernando, Surya Roca, Jorge Sancho, Álvaro Alesanco, Raquel Bailón

**Affiliations:** 1 Biomedical Signal Interpretation & Computational Simulation (BSICoS) Group, Aragón Institute of Engineering Research (I3A), IIS Aragón, University of Zaragoza, 50018 Zaragoza, Spain; rbailon@unizar.es; 2 Centro de Investigación Biomédica en Red en Bioingeniería, Biomateriales y Nanomedicina (CIBER-BBN), 28029 Madrid, Spain; 3 Communications Networks and Information Technologies (CeNIT) Group, Aragón Institute of Engineering Research (I3A), University of Zaragoza, 50018 Zaragoza, Spain; surya@unizar.es (S.R.); jslarraz@unizar.es (J.S.); alesanco@unizar.es (Á.A.)

**Keywords:** heart rate variability, wearable device, Apple Watch, RR series, validation, ANS assessment

## Abstract

Heart rate variability (HRV) analysis is a noninvasive tool widely used to assess autonomic nervous system state. The market for wearable devices that measure the heart rate has grown exponentially, as well as their potential use for healthcare and wellbeing applications. Still, there is a lack of validation of these devices. In particular, this work aims to validate the Apple Watch in terms of HRV derived from the RR interval series provided by the device, both in temporal (HRM (mean heart rate), SDNN, RMSSD and pNN50) and frequency (low and high frequency powers, LF and HF) domain. For this purpose, a database of 20 healthy volunteers subjected to relax and a mild cognitive stress was used. First, RR interval series provided by Apple Watch were validated using as reference the RR interval series provided by a Polar H7 using Bland-Altman plots and reliability and agreement coefficients. Then, HRV parameters derived from both RR interval series were compared and their ability to identify autonomic nervous system (ANS) response to mild cognitive stress was studied. Apple Watch measurements presented very good reliability and agreement (>0.9). RR interval series provided by Apple Watch contain gaps due to missing RR interval values (on average, 5 gaps per recording, lasting 6.5 s per gap). Temporal HRV indices were not significantly affected by the gaps. However, they produced a significant decrease in the LF and HF power. Despite these differences, HRV indices derived from the Apple Watch RR interval series were able to reflect changes induced by a mild mental stress, showing a significant decrease of HF power as well as RMSSD in stress with respect to relax, suggesting the potential use of HRV measurements derived from Apple Watch for stress monitoring.

## 1. Introduction

Heart rate variability (HRV) analysis is a widely accepted tool for the noninvasive assessment of autonomic nervous system (ANS) [1]. Its analysis and use are becoming increasingly common, since it is sensitive to both physiological and psychological changes [2]. Altered HRV measurements have been reported in several diseases related to ANS dysregulation, including cardiovascular diseases, such as ischemia, myocardial infarction and heart failure [1,3], metabolic diseases, such as diabetes and obesity [3], and mental disorders, such as anxiety and depression [4,5]. Moreover, HRV measurements have also been used to monitor sleep [6], stress [7,8], drowsiness [9] and exercise training [10,11,12].

Over the past 10 years, the market for health related wearable devices has grown exponentially, as well as their potential use for healthcare and wellbeing applications. The most popular and accepted ones, especially among the non-patient population, are the watch-like devices, which usually provide average heart rate (HR) estimates, derived from the pulse signal recorded at the wrist. They are based on photophethysmography (PPG), including a light source for illuminating the skin and a photodetector, which measures the intensity of the reflected light. HR estimates are based on the pulsatile changes in reflected light induced by fluctuations in blood flow every heartbeat. Although technically feasible, only a small subset of these devices allows HRV measures. As pointed out in [13], these devices usually lack from proper validation, limiting its applicability for enhancing clinical care.

Different wearable devices have been evaluated and compared for HR estimation. In [13] the Apple Watch achieved the best performance estimating HR at a one minute granularity, with median error below 3% during sitting, walking, running, and cycling. However, neither of these devices has been validated for HRV estimation. Although pulse rate variability (PRV) derived from PPG has been validated as a surrogate of HRV derived from the electrocardiogram (ECG) [14], it is not the case for PRV derived from the wrist. Only a few studies have addressed this issue, such as [15], where wrist PPG-based HRV was evaluated for Emphatica E4 (Empatica Srl, Milano, Italy) and PulseOn (PulseOn Oy, Espoo, Finland) devices, yielding reliable results only during sitting condition.

The objective of this study is to validate the Apple Watch for HRV measurement. Prior studies have only validated aggregate HR estimates [13,16,17,18,19], but its outperformance over other devices [13,20] makes it a potential candidate among wrist-worn devices for HRV measurement. Moreover, its spread and acceptance are beating the wearable market [21]. The present study sets out the comparison of RR intervals derived from Apple Watch using as reference the RR intervals derived from the Polar H7 chest belt heart rate monitor (Polar Electro Oy, Kempele, Finland), which has been already validated [22,23]. Additionally, this study includes the validation of HRV measurements, derived from the RR intervals from both devices, in two scenarios with different ANS balance: relax and metal stress.

## 2. Materials and Methods

### 2.1. Experimental Setup

A total of 20 volunteers agreed to participate in the study. All of them were apparently healthy subjects. Written informed consent was obtained from each subject. The study protocol was approved by the institutional ethics committee and was in accordance with the Declaration of Helsinki for Human Research of 1974 (last modified in 2008).

Participants were asked to abstain from caffeine and smoking prior to the test and to only consume a light meal 2 h prior to testing. The Polar H7 chest belt was placed tightly just below the chest muscles and the Apple Watch was placed tightly as well in the left wrist. Both devices were cleaned up between uses to avoid possible misreading effects. Measures were taken while participants were sitting in front of a 21″ computer screen, completing relax and mental stress test protocols. Instructions were given to minimize left arm movements, where the Apple Watch was placed.

The experimental protocol consisted of two consecutive 5-min stages, namely relax and stress stages, separated by a 1-min break. During the relax stage volunteers watched a relaxing video, consisting in relaxing music and pleasant images, which was previously used in a study on emotion recognition [24]. During the stress stage, participants performed an online version of the Stroop test (www.onlinestrooptest.com), which is an attentional test in which participants see words (names of different colors) written in colored ink and they are asked to name the color of the ink while ignoring the meaning of the word.

Raw RR data was obtained from Polar H7 ($RR_{H7}$) using Elite HRV app installed in an Android device. This app is able to export a text file containing the raw RR interval series with a precision of milliseconds. On the other hand, the Breath app was used to extract RR interval series from Apple Watch ($RR_{AW}$). Breath app, developed by Apple, is the only way at this moment (watchOS 4.2 operating system) to obtain RR raw values since Apple does not include any programing method for developers to directly access the values. This app stores the raw RR values, with a precision of centiseconds, in the user’s Personal Health Record, accessible to be exported in XML format using Apple’s Health App.

### 2.2. Synchronization and RR Matching

**Figure 1:**
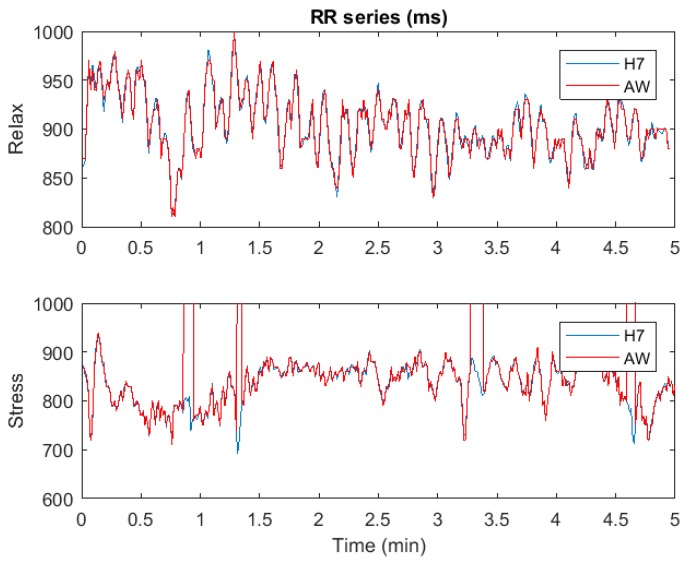
Example of the RR series for a subject in both stages: Polar H7 (blue) and Apple Watch (red). In the stress stage, there are gaps in the Apple Watch recording where no beats are detected.

Figure 1 shows an example of both RR series, $RR_{H7}$ and $RR_{AW}$. It can be seen that the RR series derived from the Apple Watch has some gaps, probably corresponding to time instants where Apple Watch proprietary algorithms have not been able to reliably detect the PPG pulses. These gaps were present in almost all Apple Watch recordings, with no apparent relation with the subject or the test stage (relax or stress). Synchronization between $RR_{H7}$ and $RR_{AW}$ was performed using the delay that maximized their cross correlation using the first 20 RR intervals, where no gaps appeared in Apple Watch recordings. All RR intervals need to be matched from both devices: we create $RR_{H7g}$ by intentionally creating the same gaps in $RR_{H7}$ than those in $RR_{AW}$. This way, both $RR_{AW}$ and $RR_{H7g}$ have the same number of samples and all RR intervals are matched. For each recording, the percentage of missing RR intervals missed with respect to the total number of RR intervals were noted.

### 2.3. Validation of RR Series

A Bland–Altman plot [25] was used to study the validity of the Apple Watch RR series, by representing both $RR_{H7g}$ and $RR_{AW}$ series. The bias, the limits of agreement (LOA, ±2*std values) and the percentage of paired RR measurements out of the LOA were also obtained.

To measure the interchangeability between both RR series, two reliability indices were used. First, Lin’s concordance correlation coefficient (CCC) determines how far the observed data deviate from the line of perfect concordance line at 45° on a square axis scatter plot [26]. Second, intraclass correlation coefficient (ICC) represents the ratio of between-sample variance and the total variance (between- and within-sample) to measure precision under the model of equal marginal distributions [27]. Lastly, the agreement was measured by an information-based measure of disagreement (IBMD), which is based on Shannon’s entropy [28]. This index equals 0 when the observers agree (no disagreement); i.e., there is no information in the differences between both methods. The agreement (A) can be quantified as A = 1−IBMD.

### 2.4. Heart Rate Variability Parameters

Heart rate variability parameters were divided in two classes: time and frequency domain indices. Since both are calculated in different ways, the gaps also are addressed differently. For the temporal domain analysis, we will derive HRV parameters from $RR_{AW}$, $RR_{H7}$ and $RR_{H7g}$, the latter created with simulated gaps as defined in Section 2.2. For the frequency domain analysis, most of the methods used for power spectral density estimation require evenly sampled series, and interpolation is usually employed to overcome the intrinsic unevenly sampled RR interval series. However, since gaps in $RR_{AW}$ and $RR_{H7g}$ are too large (from 3.3 to 10.4 s), we first filled the gaps with artificial RR intervals, created by linear interpolation. Note that, for simplicity, we use the same nomenclature, $RR_{AW}$ and $RR_{H7g}$, for temporal and frequency domain analysis, although they are slightly different: for temporal HRV parameters, the gaps are omitted and the RR intervals are concatenated ($RR_{AW}$ and $RR_{H7g}$ are shorter than $RR_{H7}$), while for frequency HRV parameters the gaps are filled with artificially created RR intervals ($RR_{AW}$ and $RR_{H7g}$ are the same length than $RR_{H7}$).

For the temporal parameters, four parameters were proposed [1]: HRM, SDNN, RMSSD and pNN50. HRM is the mean heart rate, which is obtained as the inverse of the mean heart period (mean of the RR intervals). SDNN is the standard deviation of the NN intervals (or normal RR intervals), which is a measure of the total power in the analyzed segment. A dispersion measure can be obtained by calculating the root mean-square of successive differences of adjacent intervals (RMSSD). The last parameter, pNN50, represents the percentage of pairs of adjacent intervals differing by more than 50 ms. RMSSD and pNN50 describe short-time variability. Note that whenever a gap appears in $RR_{AW}$, the corresponding intervals in $RR_{H7}$ are also removed, and the remaining RR intervals are concatenated. The difference of the intervals just before and after the gaps are not included in the calculation of RMSSD and pNN50.

For the frequency domain analysis, the heart rate variability signal was obtained following a method based on the integral pulse frequency modulation (IPFM) model described in [29]: from the RR interval series, we derived the instantaneous heart rate signal, $d_{HR}\left(n\right)$, which is a continuous and evenly sampled signal (sampled at 4 Hz). Then, we obtained the mean heart rate, $d_{HRM}\left(n\right)$, using a low pass filter up to 0.03 Hz. The variability signal, $d_{HRV}\left(n\right)$, was obtained as $d_{HRV}\left(n\right)=d_{HR}\left(n\right)-d_{HRM}\left(n\right)$. Finally, the modulating signal, which is assumed to represent the ANS modulation on the sinoatrial node, was obtained as $\hat{m}\left(n\right)=d_{HRV}\left(n\right)/d_{HRM}\left(n\right)$ [30].

The Welch periodogram was applied to estimate the spectral properties of the HRV signals ${\hat{m}}_{AW}\left(n\right)$, ${\hat{m}}_{H7}\left(n\right)$ and ${\hat{m}}_{H7g}\left(n\right)$ (derived from $RR_{AW}$, $RR_{H7}$ and $RR_{H7g}$, respectively), using a Hamming window with a length of 150 s with an overlap of 120 s. Low frequency (LF) and high frequency (HF) powers, $P_{LF}$ and $P_{HF}$ respectively, were obtained integrating the power in their bands (LF: 0.04–0.15 Hz, HF: 0.15–0.4 Hz) [1].

### 2.5. Statistical Analysis

None of all HRV parameters followed a normal distribution (tested with a Kolmogorov test). To compare HRV parameters, a paired Wilcoxon test was applied. Then we performed 3 different comparisons: (1) we analyzed the influence of the gaps by comparing HRV parameters derived from $RR_{H7}$ and $RR_{H7g}$; (2) we compared HRV parameters derived from $RR_{AW}$ and $RR_{H7g}$; and (3) HRV parameters derived from $RR_{AW}$ were compared in relax vs. stress stage to evaluate the ability of those parameters to reflect ANS response to stress, using changes in HRV parameters derived from $RR_{H7}$ as the reference ANS response. The difference was considered to be significantly different from zero when *p* < 0.05.

Distribution of HRV parameters are shown using boxplots: on each box, the central mark indicates the median, and the bottom and top edges of the box indicate the 25th and 75th percentiles, respectively. The whiskers extend to the most extreme data points not considered outliers, and the outliers are plotted individually using the ‘+’ symbol. An outlier is defined as a point that falls more than 1.5 times the interquartile range (difference between 75th and 25th percentiles) above the 75th percentile or below the 25th percentile.

## 3. Results

### 3.1. Validity of RR Series

A total of 206 gaps were found in the Apple Watch recordings, equivalent to 1321 missing RR intervals (around 10% of total intervals). On average, each recording presents 5 gaps, with a length of 6 s per gap. The minimum length found was 3.3 s and the maximum length was 10.4 s. No differences in the number or length of these gaps were found between relax and stress recordings.

**Figure 2:**
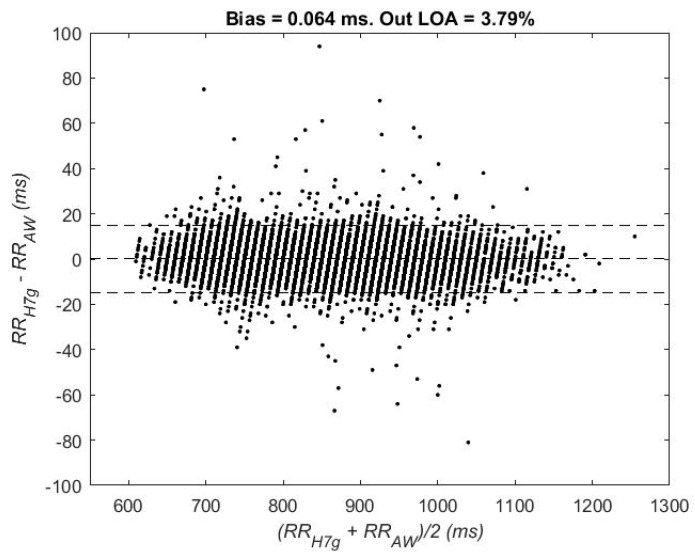
Bland-Altman plot: $RR_{H7g}\;\mathrm{vs}.\;RR_{AW}$. Mean of the difference of the RR series ±2*std values (limits of agreement, LOA).

Figure 2 shows the Bland–Altman plot which evaluates the RR interval discrepancies between Polar H7 and Apple Watch measurements and the stability across the different values of the intervals. A total of 12,109 paired RR intervals were used from both relax and stress stages. The bias (central horizontal line) was 0.06 ms. The LOA (upper and lower lines) contained 96.21% of the intervals. No visual differences were found in the discrepancies between lower and higher RR intervals.

Table 1 shows the mean values of the RR intervals for each stage, as well as the reliability and agreement indices. While slightly lower in the stress stage, these indices show excellent reliability and agreement between the measurements in both stages. Bias and LOA are also shown for relax and stress stages, which are very similar to those obtained with the combined data. The last row shows the mean of the percentage of missing RR intervals in $RR_{AW}$.

### 3.2. HRV Parameters: Temporal Domain

**Figure 3:**
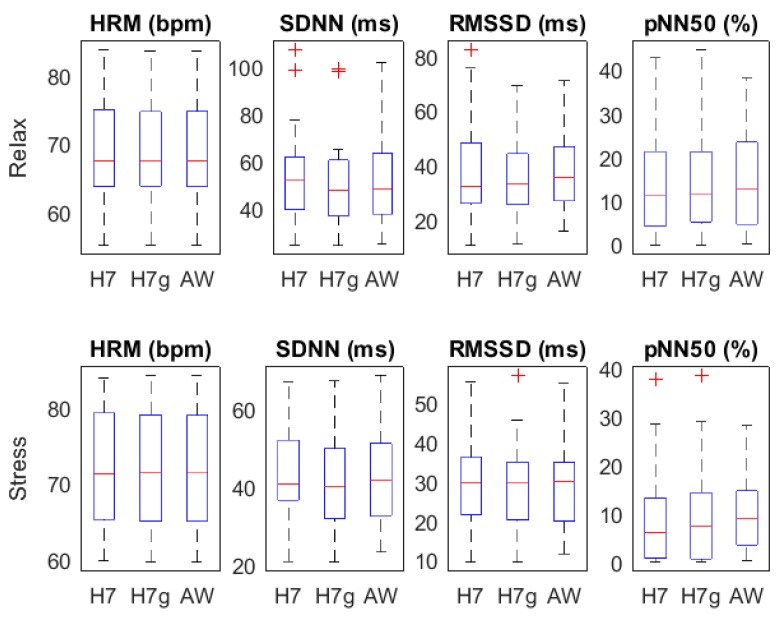
Heart rate variability (HRV) parameters: time domain.

Figure 3 shows the temporal parameters for both relax and stress stages. No significant differences were found in any parameter neither between $RR_{H7}$ and $RR_{H7g}$, nor between $RR_{H7g}$ and $RR_{AW}$.

### 3.3. HRV Parameters: Frequency Domain

**Figure 4:**
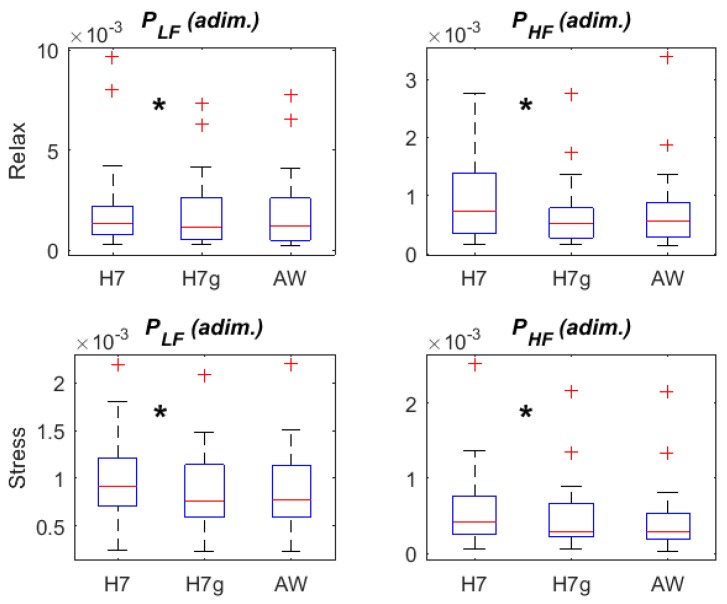
HRV parameters: frequency domain, derived from $RR_{H7}$, $RR_{H7g}$ and $RR_{AW}$. * denotes significant differences (*p* < 0.05) between adjacent boxplots. Adim refers to adimensional units.

Figure 4 shows the frequency HRV parameters derived from $RR_{H7}$, $RR_{H7g}$ and $RR_{AW}$ in relax and stress stages. Both $P_{LF}$ and $P_{HF}$ show significant differences (marked with an asterisk between the boxplots) in $RR_{H7g}$ when comparing to $RR_{H7}$, being lower in the presence of the gaps. When comparing HRV parameters derived from $RR_{AW}$ and $RR_{H7g}$, neither $P_{LF}$ nor $P_{HF}$ presented significant differences.

### 3.4. HRV Parameters: Relax vs. Stress

**Figure 5:**
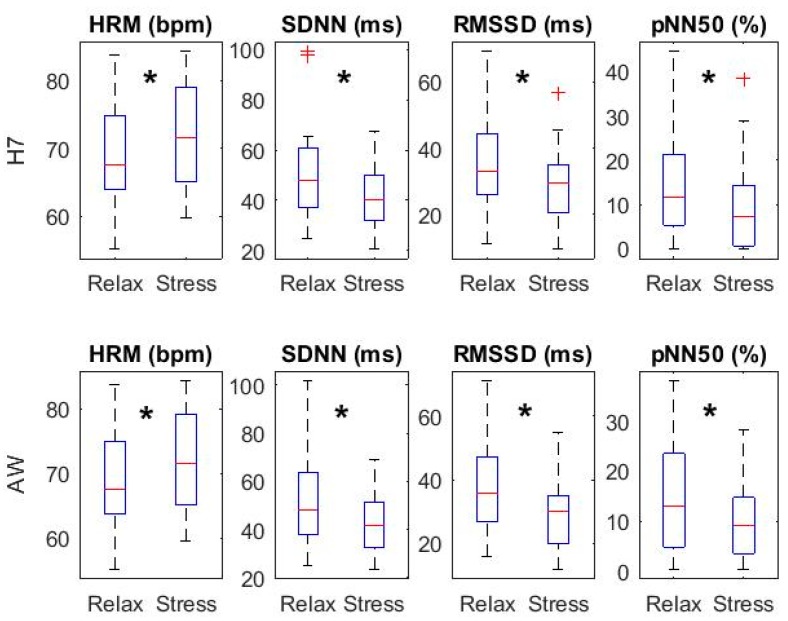
HRV parameters: temporal domain (Relax vs. Stress). * denotes significant differences (*p* < 0.05) between adjacent boxplots.

**Figure 6:**
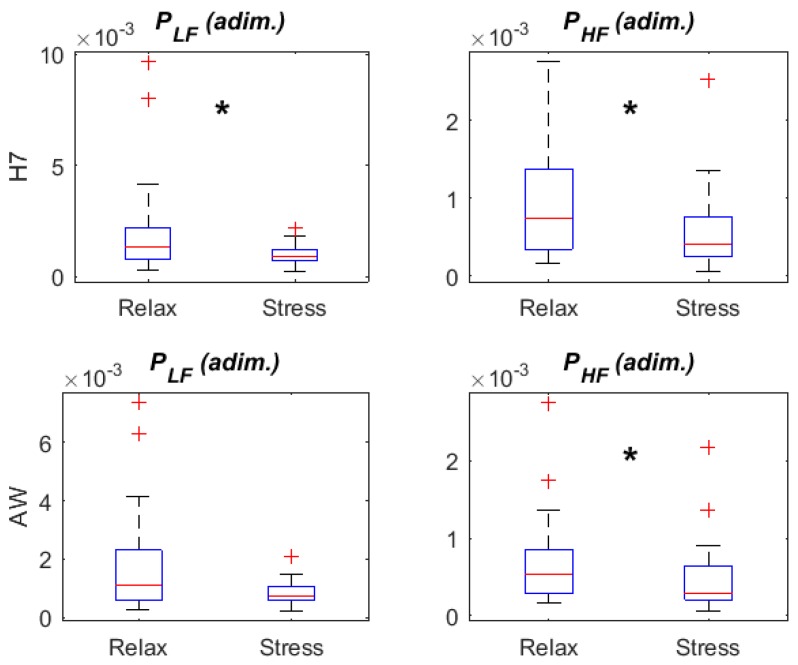
HRV parameters: frequency domain (Relax vs. Stress). * denotes significant differences (*p* < 0.05) between adjacent boxplots. Adim refers to adimensional units.

Figure 5 and Figure 6 display HRV parameters derived from $RR_{AW}$ and $RR_{H7}$ in relax vs. stress stages. All temporal HRV parameters (HRM, SDNN, RMSSD and pNN50) presented changes from relax to stress in both Apple Watch and Polar H7 recordings: an increase in HRM and a decrease in the rest of parameters. We observed a decrease in $P_{LF}$ in stress with respect to relax stage, being only statistically significant in Polar H7 recordings (*p* = 0.030 for Polar H7, *p* = 0.065 for Apple Watch). A significant decrease was found in both devices when comparing $P_{HF}$.

## 4. Discussion

This is the first study to validate HRV measurements using an Apple Watch to the authors’ knowledge. Many of the wrist-worn devices aimed at assessing well-being, stress, and fitness provide average HR values, derived from a pulse signal, instead of HRV. Fitbit, Jawbone, TomTom, Garming, Withing, Samsumg, among others, commercialize such devices. However, although changes in average HR are mainly induced by ANS and can be significantly different in some physio-pathological situations, it cannot be considered a measure of autonomic function [3,31]. Moreover, there are situations where altered ANS function is manifested in HRV but not in HR changes, such as in depressed patients with respect to controls [32] or in exercise contexts [33,34].

In order to validate HRV measurements derived from an Apple Watch, in this work an experimental study was conducted on young healthy volunteers subjected to mild relax and stress. HRV measurements derived from Apple Watch were compared to those obtained from a validated heart rate monitor, in particular Polar H7 chest band. Several works in the literature have already validated, using as reference the RR interval series derived from a synchronous ECG, the RR interval series given by different models of Polar devices: S810 [35,36], RS800 [37,38], and V800 with a H7 chest belt [22,23].

Regarding the RR intervals series, which are the basis for any HRV analysis, the bias was almost 0 and most pairs of RR intervals (around 96%) were within the limits of agreement in the Bland Altman plot. These results are consistent when studying both stages separately. Moreover, the discrepancy between Apple Watch and Polar H7 is similar with no dependency of the heart rate. The RR series present excellent reliability and agreement when analyzing matched pairs of RR intervals. These results agree with other studies for Apple Watch [17,18] and other heart rate watches [13,16,19].

However, it is important to note the existence in Apple Watch measurements of missing beats. Around 10% of the beats could not be detected by the Apple Watch in both the relax and stress stages. This could be not problematic if they were isolated beats, but they usually were consecutive beats, which lead to gaps in the RR series. The shorter gaps were longer than 3 s, being easily identifiable as abnormally large RR intervals. The origin of these gaps could be varied, from bad skin contact to fast arm movement, but ultimately, we cannot conclude anything about these gaps because we do not have direct access to the algorithms used by the Apple Watch. In [18], they also reported missing values in the Apple Watch, with the proportion of heart rate values actually measured by the Apple Watch decreasing with increasing exercise intensity. They also reported that these missing heart rate values were higher in the first minute of exercise (between 20 and 40% of RR intervals), particularly at higher intensities of exercise. In our data, however, we did not find more missing beats in the stress stage compared to the relax stage, and the total missing RR intervals was about 10% of total intervals.

These missing beats are important when extracting HRV parameters in the frequency domain, since the RR interval series needs to be interpolated in the gaps. This might influence HRV frequency parameters. To study the influence of these gaps in frequency parameters $P_{LF}$ and $P_{HF}$, we created $RR_{H7g}$ by intentionally creating gaps in the $RR_{H7}$ series, removing the missing RR intervals found in $RR_{AW}$. This analysis showed that both $P_{LF}$ and $P_{HF}$ were significantly lower in the presence of simulated gaps. However, when comparing HRV frequency parameters derived from $RR_{AW}$ and from $RR_{H7g}$, no significant difference was found, suggesting that differences between HRV parameters derived from RR series provided by Apple Watch and Polar H7 are indeed due to the presence of gaps in the Apple Watch series, rather than to the different signal from which they are derived or to the different time resolution. The effect of gaps on HRV temporal parameters was also investigated, but no significant differences were found, suggesting that these parameters are less sensitive to the presence of gaps.

Despite these differences, HRV measurements derived from Apple Watch were still able to reflect the ANS response induced by the stress stage, replicating most of the trends observed in HRV measurements derived from the Polar H7. Induced mental stress resulted in a significant decrease in $P_{HF}$ with respect to relax (in both the Apple Watch and Polar H7 measures, note that there were no gaps in the Polar H7 recordings), suggesting the inhibition of parasympathetic stimulation, as already reported in numerous studies [8,39]. A similar conclusion can be extracted with the decrease in RMSSD parameter in the stress phase, being this parameter associated to the parasympathetic activity. Although not shown in the results, the ratio between LF and HF powers (*R*), as well as the normalized LF power ($P_{LFn}$), were also obtained. Comparing relax and stress, these parameters have shown in other studies a significant increase during stress [8], which means a predominance of the sympathetic activity. In this work, however, the increase found in *R* and $P_{LFn}$ was not significant even in the Polar H7 measures, possibly due to the fact that the level of stress was moderate: see the moderately low maximum HR achieved in the stress stage (around 100 bpm). Still, the significant decrease in $P_{HF}$ was evident using both the Polar H7 and the Apple Watch, supporting the potential use of the Apple Watch for stress monitoring.

However, one of the main limitations of using the Apple Watch to obtain and use HRV measures is that, at this moment, the RR series is only available through the use of the Apple Breath App in an indirect way (exporting the measured values using the Health App, where all Health-related data from the user is stored). Apple does not provide within the watch OS 4.2 SDK any function to access raw RR measures. Thus, developers are not able to develop an App that takes advantage of the potential shown by Apple Watch in HRV monitoring. Besides, Breath App stops when repeated motion is detected. This does not allow us to validate HRV measurements in low or moderate exercise. Nevertheless, this SDK limitation can be removed by Apple in a further SDK release. This inclusion would leverage the development of Apps able to monitoring stress conditions, among other things, by the developer’s community.

## 5. Conclusions

Heart rate variability parameters extracted from an Apple Watch device have been validated using a Polar H7 band as a reference during relax and mental stress in 20 healthy volunteers. Reliability and agreement coefficients were computed for the RR interval series provided by both devices in relax and stress stages, achieving very good results (reliability and agreement > 0.9). No significant differences were found when comparing temporal HRV parameters (SDNN, RMSSD, pNN50 and HRM) derived from the RR interval series provided by both devices. However, frequency HRV parameters LF and HF powers were significantly different when derived from the Apple Watch, due to the appearance of gaps in the RR series, both in relax and stress stages. Nonetheless, a decrease in the HF power (and in the RMSSD parameter) was observed in stress with respect to relax stage when using both devices, which supports the potential use of the Apple Watch for stress monitoring.

## Figures and Tables

**Table 1:** Validity of RR series from Apple Watch. CI = Confidence interval.

	RELAX	STRESS
Mean (SD) H7 RR intervals (ms)	869.28 (114.01)	834.78 (97.43)
Mean (SD) AW RR intervals (ms)	869.23 (114.39)	834.70 (97.84)
CCC (90% CI)	0.989 (0.981, 0.998)	0.977 (0.970, 0.985)
ICC (90% CI)	0.989 (0.984, 0.996)	0.982 (0.977, 0.987)
A (90% CI)	0.993 (0.987, 0.999)	0.983 (0.975, 0.991)
Bias (Out LOA)	0.049 (3.29%)	0.078 (3.93%)
Mean (SD)% missed RR intervals	10.98 (9.78)	9.45 (7.30)
